# PReS-FINAL-2314: Anti-TNF alpha therapy for refractory childhood takayasu arthritis

**DOI:** 10.1186/1546-0096-11-S2-P304

**Published:** 2013-12-05

**Authors:** B Koren, Š Blazina, N Toplak, D Ključevšek, T Avčin

**Affiliations:** 1 University medical centre, Maribor, Slovenia; 2 University medical centre, Ljubljana, Slovenia

## Introduction

Takayasu arteritis (TA) is a rare chronic granulomatous vasculitis of large vessels. Initial symptoms and signs are usually non-specific, therefore a high index of suspicion is needed to make a timely and correct diagnosis.

## Objectives

To review our experience with treatment of children with TA.

## Methods

We analysed patients data.

## Results

In our centre we are currently treating two adolescents with TA. Patient 1 presented with arthralgia, anaemia and fatigue. Patient 2 demonstrated leg claudication. None of them had hypertension. In both of them vascular bruits and elevated inflammatory markers were present. Diagnosis was confirmed by magnetic resonance angiography (MRA). Both patients were initially treated with pulse methylprednisolone followed by a high dose oral corticosteroid in combination with methotrexate. Both patients achieved transient clinical and laboratory improvement with decreased inflammatory parameters after initial treatment. By tapering steroid therapy inflammatory parameters started to rise. Patient 2 also demonstrated less palpable leg pulses and persistent leg claudication. Follow-up MRA imaging demonstrated only partial improvement with persistent vascular changes in both patients. Because of incomplete disease control both patients were started on anti-tumor necrosis factor (TNF) alpha therapy with infliximab which resulted in good clinical and laboratory response with decreased inflammatory parameters.

## Conclusion

According to our experience anti-TNF alpha therapy appears to be a successful treatment approach in pediatric patients with refractory TA.

## Disclosure of interest

None declared.

